# A Microcosm Experiment Reveals the Temperature-Sensitive Release of *Mucochytrium quahogii* (=QPX) from Hard Clams and Pallial Fluid as a Stable QPX Reservoir

**DOI:** 10.3390/microorganisms12020241

**Published:** 2024-01-24

**Authors:** Sabrina Geraci-Yee, Jackie L. Collier, Bassem Allam

**Affiliations:** School of Marine and Atmospheric Sciences, Stony Brook University, Stony Brook, New York, NY 11794, USAjackie.collier@stonybrook.edu (J.L.C.)

**Keywords:** Quahog Parasite Unknown, *Mercenaria mercenaria*, QPX disease, commensal, labyrinthulomycetes, thraustochytrid, host-pathogen-environment interactions

## Abstract

*Mucochytrium quahogii*, also known as QPX or Quahog Parasite Unknown, is the causative agent of QPX disease in the hard clam (*Mercenaria mercenaria*). Host–pathogen–environment interactions between *M. quahogii*, the hard clam, and temperature were explored in a microcosm experiment. Hard clams were housed in individual tanks with sterile seawater under two temperature regimes: low (13 °C) temperature, which is thought to be optimal for QPX disease development, and high (20 °C) temperature, which has been shown to promote “healing” of QPX-infected clams. Hard clam tissue, pallial fluid, seawater, and shell biofilms were collected and assayed for *M. quahogii*. The release of *M. quahogii* from naturally infected live hard clams into seawater was detected only in the low temperature treatment, suggesting that temperature influences the release of potentially infectious cells. *M. quahogii* was commonly found in hard clam pallial fluid, even after 9 weeks in the lab, suggesting pallial fluid is a stable reservoir of *M. quahogii* within its primary host and that *M. quahogii* is not a transient component of the hard clam microbiota. Overall, results support a host-specific relationship and that *M. quahogii* is a commensal member of the hard clam microbiota, supporting its classification as an opportunistic pathogen.

## 1. Introduction

Mortality caused by disease in wild and cultured hard clams (*Mercenaria mercenaria*) was considered rare until 1989, when an infectious agent causing mass mortalities in Canada was identified and named Quahog Parasite Unknown (QPX) [1]. In the United States, the first major outbreak of QPX disease occurred in 1992 in cultured hard clams of Massachusetts, with mortality approaching 80% of marketable stocks [2], with additional mass mortality events reaching up to 95% of populations reported throughout the northeast Atlantic coast in New York, New Jersey, Rhode Island, and Virginia [3,4,5,6,7]. Although QPX had been isolated from hard clams and cultured since the 1990s, it was not until 2021 that it was formally described as *Mucochytrium quahogii* (S. Geraci-Yee et B. Allam) [8], and it is still commonly referred to as QPX. 

*M. quahogii* belongs to the thraustochytrids, a diverse family of labyrinthulomycetes, which are a common and abundant group of marine fungus-like protists. The labyrinthulomycetes are classified as stramenopiles (heterokonts) and comprise four monophyletic groups: thraustochytrids, oblongichytrids, aplanochytrids, and labyrinthulids [9]. Most labyrinthulomycetes are nonpathogenic osmoheterotrophs, functioning ecologically as fungus-like saprobes, involved in the decomposition of organic matter [10]. In nature, the labyrinthulomycetes are associated not only with decaying substrates [11], but they are also commonly found in living marine metazoans and metaphytes [10,12]. The regular association of labyrinthulomycetes with apparently healthy marine plants and animals suggests that these relationships are generally mutualistic or commensal in nature. However, in addition to *M. quahogii* and the hard clam, other labyrinthulomycetes have been reported as opportunistic parasites in a variety of marine organisms [13,14]. Research into labyrinthulomycete pathogens has revealed their broad distribution in the marine environment, association with apparently healthy individuals, and opportunistic pathogenesis in response to changing host–microbe–environment interactions [15,16,17,18]. However, the question remains—are they avirulent commensals of their hosts or environmentally acquired pathogens? This study seeks to answer this question for the case of *M. quahogii* and the occurrence of QPX disease in the hard clam.

If *M. quahogii* is an environmentally acquired pathogen, then it may gain access to the hard clam pallial tissues—where QPX infections appear to originate—during seawater pumping and filtering [2,4,19,20,21]. This requires the presence of infectious *M. quahogii* propagules in the environment, and indeed *M. quahogii* has been detected at low abundance but widespread throughout sediment, seawater, and other components of the hard clam habitat [16,20,22,23]. Whether *M. quahogii* lives independently in the environment, or is released at a sufficient rate from the hard clam (or other) hosts to maintain its observed distribution, is unknown. The natural transmission mechanism and infectious stage(s) of *M. quahogii* also remains unclear, although infection experiments have been successful in causing disease in naïve clams through injection of cultured, vegetative *M. quahogii* cells [24]. It should be noted that clam genetic background and prevailing environmental conditions have been shown to represent determinant factors for disease establishment and development. For instance, QPX-infected clams are able to heal from QPX disease through encapsulation and destruction of parasite cells by clam hemocytes. This immune response by clam hemocytes to *M. quahogii* is thought to be temperature-dependent, with evidence from the field showing the response is stronger in the summer than in the spring and fall [1,2,3,21]. Laboratory studies have confirmed the influence of temperature on QPX disease development, which is faster and more severe at 13 °C compared to higher temperatures (21 and 27 °C), with QPX-infected hard clams displaying healing of existing QPX infections [25,26].

In a recent survey of wild hard clams, it was determined that “healthy” hard clams (no detection of *M. quahogii* via quantitative PCR in hard clam tissue) contained *M. quahogii* in their pallial fluid [16]. Since *M. quahogii* was prevalent (73% of clams) and the dominant labyrinthulomycete (70% of clams contained only *M. quahogii*) in hard clam pallial fluid, *M. quahogii* either represents a (1) common transient inhabitant from seawater or (2) a more permanent resident of the pallial fluid microbiota. The pallial fluid, which is the fluid within the mantle cavity of bivalves that surrounds the pallial organs, is usually considered continuous with seawater, as vast quantities of seawater and associated microbes are pumped into the cavity. Within the pallial cavity, the formation and storage of pseudofeces, which have been shown to harbor *M. quahogii*, is thought to be involved in the infection process [20,27]. In this framework, pallial fluid and its associated mucus and pseudofeces may represent an important QPX reservoir.

The purpose of this study was to determine if *M. quahogii* (1) can be released from naturally QPX-infected hard clams and (2) is a transient or more permanent resident of the hard clam microbiota, including pallial fluid and shell biofilms. We performed a laboratory microcosm experiment, in which hard clams were housed under two temperature regimes: 13 °C, which is thought to be optimal for QPX disease development, and 20 °C, which has been shown to promote “healing” of QPX-infected clams [25,26,28]. The microcosm experiment used filtered and UV-sterilized seawater, so that the only *M. quahogii*—and other labyrinthulomycetes—detected must have been introduced with the hard clams. We hypothesized that *M. quahogii* release from naturally infected hard clams is common, regardless of temperature, and that *M. quahogii* is a long-term resident of the hard clam microbiota, characteristic of a commensal relationship with the hard clam. The microcosm experiment revealed that *M. quahogii* was prevalent in hard clam pallial fluid, even after 9 weeks in the microcosm, suggesting that *M. quahogii* is a stable, long-term resident of the hard clam microbiota and supporting commensalism between *M. quahogii* and the hard clam.

## 2. Methods

Thirty-eight hard clams (littleneck/top neck size, 2.5–5 cm across the hinge) were obtained from a heavily QPX-impacted clamming site in Barnstable, Massachusetts (MA) in 2014. Clams were lightly washed and stored in QPX-free 0.2 µm filtered, UV-sterilized natural seawater (collected from Stony Brook Harbor, New York; from here on referred to as “sterile seawater”) for 1 week for acclimation and reduction of surface-bound and/or gut-transiting microorganisms, including *M. quahogii* (=QPX). A subset of these clams (*n* = 16) was assessed for QPX disease by quantitative PCR (qPCR) in tissue samples (mantle tissue homogenate) pre-experiment (time 0), using the updated diagnostic qPCR assay [29]. After the acclimation period, 22 clams were placed in individual 3 L tanks filled with sterile seawater aerated using air stones. A total of 11 tanks were held in a water bath at 13 °C and 11 tanks were held at 20 °C. The experiment ran for 9 weeks. Clams were monitored daily for mortality or signs of QPX disease (gaping). Clams were fed once weekly with 1 mL of commercial shellfish diet (Shellfish Diet 1800, Reed Mariculture, Campbell, CA, USA). Since the experiment began with sterile seawater, the only source of labyrinthulomycetes should be from the hard clam microbiota. Seawater, including clam feces, pseudofeces, and mucoid secretions released into seawater, was mixed within each individual tank prior to sampling. A total of 1 L of seawater was removed from a subset of tanks (Tanks #1-6 at 13 °C and Tanks #12-17 at 20 °C) during week 1, 2, 5, and 7, which was replaced with new sterile seawater. From the 1 L of seawater removed, 400 mL to 1 L was filtered onto a 0.4 µm polycarbonate filter (or two filters if necessary, to meet the minimum of 400 mL) under low pressure (<5 in Hg) and the filter(s) was stored at −80 °C until DNA extraction. At the end of the experiment, seawater was collected (9-week sample), biofilms growing on the clam shell were swabbed (sterile cotton-tipped applicator), pallial fluid was collected, and QPX disease diagnostics were performed [16,29]. Hard clam mantle tissue homogenate samples were stored at −80 °C until extracted with the NucleoSpin Genomic DNA Tissue kit (Macherey-Nagel, Inc., Bethlehem, PA, USA) and assayed by qPCR [29]. Pallial fluid, seawater, and shell swabs were stored at −80 °C until DNA extraction with the MO BIO PowerSoil DNA Isolation kit (Qiagen, Hilden, Germany) and quantification by PicoGreen (Molecular Probes, Eugene, OR, USA). QPX was assayed in pallial fluid using qPCR [29] (same assay as tissue samples) and in seawater and shell swabs using a nested qPCR (nqPCR) assay described previously [30]. For hard clam tissue and pallial fluid samples, weighted prevalence (WP) was determined based on the sum of QPX load rated on an intensity scale (Supplementary Table S1) for each individual clam, divided by the number of clams assayed for each sampling event or cohort [16,29]. As previously established, for the qPCR assay (hard clam tissue and pallial fluid), samples that had a C_q_ value and appropriate melt curve peak temperature (T_m_) but were below the limit of detection (LOD) of the assay were considered positive and denoted “BLD” (below the LOD), since they were not truly negative but also could not be accurately quantified [16,29]. Differences between clam sample types and temperature treatments were assessed using Fisher exact test or Chi-square test using the native stats package in R [31,32]. Data visualization was performed using R package “ggplot2” [33] or Microsoft Excel.

The labyrinthulomycete composition was determined by sequencing of PCR products from pallial fluid, seawater (9-week sample), and shell swabs collected at the end of the experiment. DNA was extracted with the MO BIO PowerSoil DNA Isolation kit and amplified with labyrinthulomycete-specific primers LABY-A and LABY-Y, as previously described [16], except 3 µL of template DNA was used for pallial fluid samples and 1 µL of template DNA was used for the seawater and shell swab samples. For reactions successful in their first attempt, PCR products were cleaned using ExoSAP-IT PCR Product Cleanup Reagent (Applied Biosystems, Foster City, CA, USA), followed by Sanger sequencing. Most pallial fluid samples and some shell swabs and seawater samples that did not produce a PCR product were amplified again with more template DNA (6 µL for pallial fluid samples, 3 µL for seawater samples, and 1 µL for shell swab samples) with 40 cycles and an anneal temperature reduced to 48 °C in an effort to overcome the bias of primer LABY-Y against several groups of labyrinthulomycetes, particularly the thraustochytrids. These reactions normally produced several bands and the expected ~430 bp band was gel-extracted using the Wizard SV Gel and PCR Clean-Up System (Promega, Madison, WI, USA) and Sanger sequenced. Similar downstream analyses were performed as previously described [16]. Briefly, samples that contained a mix of sequences were deconvoluted using “Base-Calling Algorithm with Vocabulary” (BCV) [34]. All output sequences, referred to as clusters, were used in downstream analyses (usually 1–3 clusters per chromatogram). Based on the NCBI GenBank Blastn result and phylogenetic analysis, each original sequence or BCV cluster was assigned a labyrinthulomycete phylogenetic group (i.e., labyrinthulid, aplanochytrid, oblongichytrid, thraustochytrid, or *M. quahogii*/QPX). A value of 1 was assigned for samples that had clean chromatograms, while for the BCV clusters, the expected portion of the mixed sample generated by BCV was used. In the phylogenetic analysis, there were 134 sequences, comprised of 69 sequences/clusters from the microcosm samples (*n* = 58 sequenced amplicons) and 65 reference sequences in the alignment, which was used to construct a Maximum Likelihood phylogenetic tree based on 397 positions. Data visualization was performed using R package “ggplot2” [33].

## 3. Results

**Figure 1:**
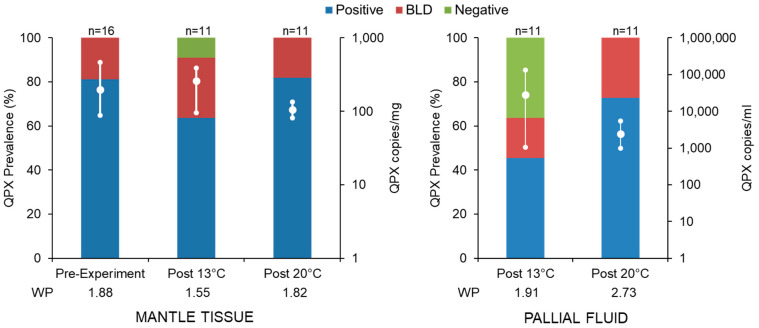
*M. quahogii* (=QPX) prevalence determined by the qPCR assay from hard clam mantle tissue and pallial fluid. BLD represents samples that were positive but below the limit of detection of the assay (non-quantifiable). The mean and range (high-low lines) of QPX concentration in each treatment is shown on the secondary axis for positive, quantifiable samples on a log10 scale. On the *x*-axis under each treatment is the weighted prevalence (WP).

At time 0 (pre-experiment), *M. quahogii* (=QPX) was found in 100% of the Massachusetts (MA) hard clam tissue samples (*n* = 16) with a mean QPX concentration of 198 copies/mg ± 107 (standard deviation; SD), confirming that these clams were naturally infected with QPX at a “light” intensity with a weighted prevalence (WP) of 1.88 (Figure 1; Supplementary Table S2). There was no mortality of hard clams throughout the experiment. At the end of the 9-week experiment, QPX was found in 100% of clam tissue samples in the 20 °C treatment with a WP of 1.82 and 91% in the 13 °C treatment with a WP of 1.55, with an overall QPX infection prevalence of 95.5%. There was a significant decrease in QPX concentration in tissue from pre-experiment (time 0) to the end of experiment in the 20 °C treatment (Wilcox rank sum test, *p* = 0.005) from an average of 198 copies/mg to 105.5 copies/mg, while the 13 °C treatment did not show a significant difference (*p* = 0.3). There was no difference in QPX prevalence between the two temperature treatments (Fisher exact test, *p* = 1).

**Figure 2:**
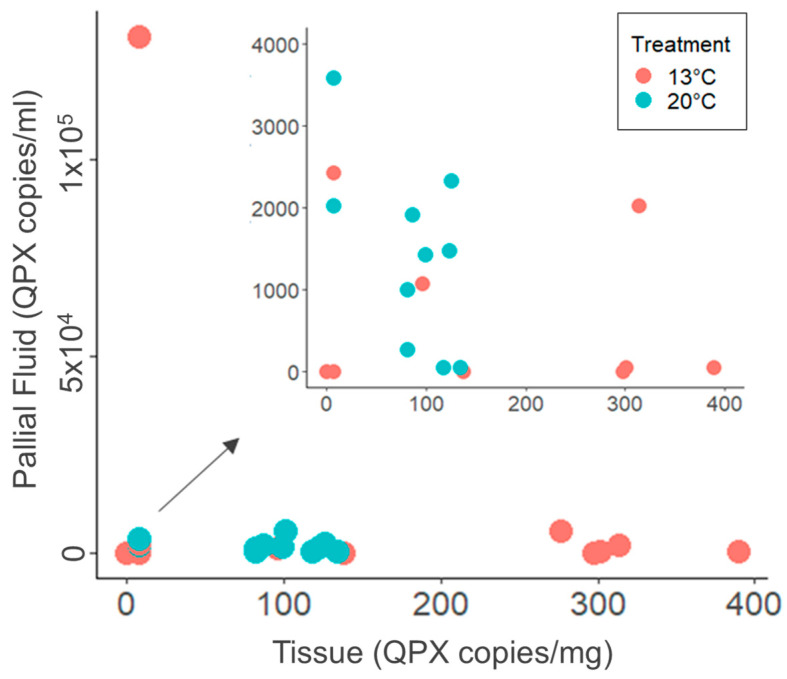
*M. quahogii* (=QPX) concentration in hard clam pallial fluid and mantle tissue for each individual hard clam by treatment. For visualization, BLD samples were assigned the value of 7.5 copies/mg tissue and 50 copies/mL pallial fluid.

After 9 weeks in laboratory microcosms, QPX was found in 82% (*n* = 18) of all pallial fluid samples with a WP of 2.32 (Figure 1; Supplementary Table S2). From each clam, approximately 1 mL (±0.4 mL standard deviation) of pallial fluid was collected, with a range of 0.1 to 1.5 mL. Most pallial fluid samples did not contain pseudofeces (*n* = 17, 77%; Supplementary Table S3), and there was no difference in QPX prevalence between pallial fluid samples with or without pseudofeces (Fisher exact test, *p* = 0.532). Additionally, there was no difference in QPX concentration between samples with and without pseudofeces (Wilcox rank sum test, *p* = 0.5). In the 20 °C treatment, 100% of pallial fluid samples were QPX-positive, while only 64% of samples in the 13 °C treatment were QPX-positive (Figure 1). There was no significant difference in QPX concentration between the two treatments (Wilcoxon rank sum test, *p* = 0.28). Additionally, there was no significant difference in QPX prevalence in pallial fluid between the two temperature treatments (Fisher exact test, *p* = 0.09). While there was no significant difference between the number of QPX-positive samples (BLD samples were considered positive) in clam tissue and pallial fluid samples at the two temperature treatments (Chi-square test 2 × 2, X^2^ = 0.3004, *p* = 0.584), tissue and pallial fluid samples from the same clam that were either negative or BLD for one sample type were usually positive and quantifiable for the other sample type (Figure 2).

**Figure 3:**
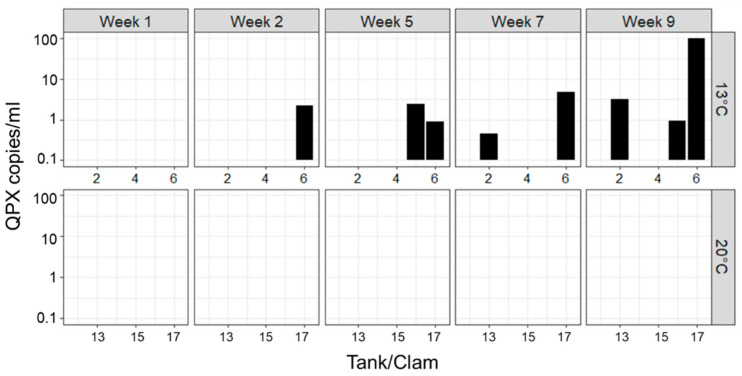
*M. quahogii* (=QPX) detected in initially sterile seawater over the course of the microcosm experiment for the subset of tanks examined (*n* = 12, 6 from each treatment) on a log10 scale. QPX was only detected in the 13ºC treatment, beginning at week 2.

**Figure 4:**
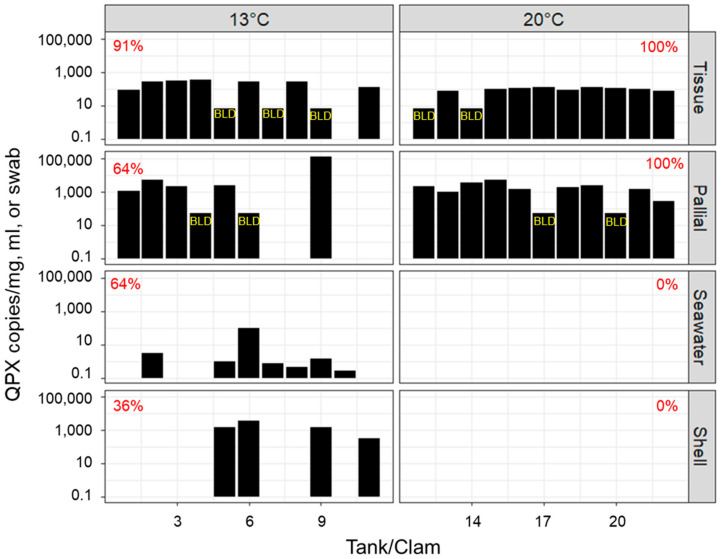
*M. quahogii* (=QPX) detected in hard clam tissue, pallial fluid, seawater, and shell swabs by qPCR/nqPCR by individual tank/clam at the end of the microcosm experiment (9 weeks) for all tanks/clams (*n* = 22, 11 from each treatment) on a log10 scale. Tank/clam #1-11 represent the 13 °C treatment and #12-22 represent the 20 °C treatment. The percentage of positive samples by treatment and sample type is shown in red for each panel. BLD samples (qPCR only) were assigned 10% of the sample type LOD: 7.5 copies/mg for tissue and 50 copies/mL for pallial fluid for visualization and are labeled in yellow.

During the course of the 9-week experiment, QPX in seawater samples was assayed from a subset of the tanks five times. QPX was only detected in tanks from the 13 °C treatment, beginning at 2 weeks, while it was never detected in the 20 °C treatment (Figure 3). At the end of the experiment, all tanks/clams were assessed for QPX in seawater and shell swabs (Figure 4). QPX was never detected in seawater or shell swabs in the 20 °C treatment, while 64% of seawater samples and 36% of shell swab samples were QPX-positive in the 13 °C treatment. *M. quahogii* concentrations assessed by qPCR and nqPCR assays sampled at the end of the microcosm experiment are summarized in Supplementary Table S4.

**Figure 5:**
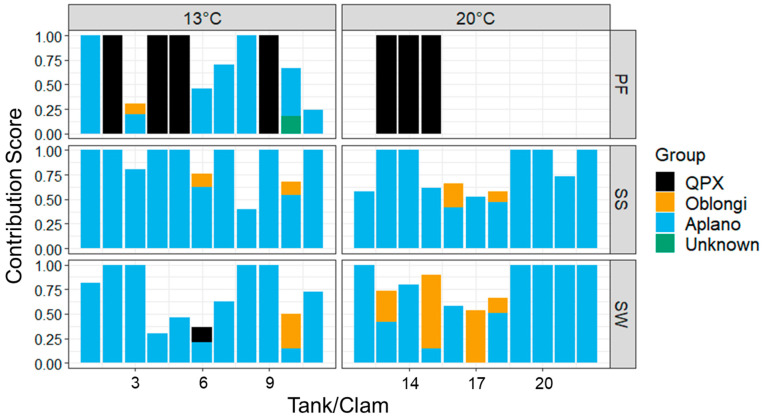
Labyrinthulomycete composition of hard clam pallial fluid (PF), shell swabs (SS), and seawater (SW) at the end of the microcosm experiment (9 weeks) for individual tanks/clams under the two temperature treatments. The y-axis represents the expected BCV contribution score for mixed sequences that were deconvoluted, while sequences containing a single dominant product were assigned the value of 1. QPX = *M. quahogii*; Oblongi = oblongichytrid; Aplano = aplanochytrid; Unknown = unknown labyrinthulomycete.

PCR products were produced by the general labyrinthulomycete primers LABY-A/LABY-Y from all seawater and shell swab samples at both temperature treatments and pallial fluid samples from 13 °C, while only 3 out of 11 pallial fluid samples from the 20 °C treatment produced PCR products, despite being 100% positive for *M. quahogii*. Of the 58 sequenced LABY-A/LABY-Y PCR products, 30 had a single sequence (clean chromatogram) and were directly used in downstream analyses, while 28 sequences were mixed and deconvolution with BCV resulted in 39 clusters. Sequenced products or BCV clusters were identified using Blastn, and phylogenetic analysis was used to validate the labyrinthulomycete group assignment (Figure 5; Supplementary Files S1 and S2). A total of 50% of the pallial fluid samples contained QPX (*n* = 7) and only QPX was found in the 20 °C treatment (*n* = 3), while the 13 °C treatment pallial fluid samples contained QPX, aplanochytrids, an oblongichytrid, and one unidentified labyrinthulomycete. In the shell swab and seawater samples, aplanochytrids were dominant, found in every sample, followed by oblongichytrids, with one sample containing an oblongichytrid and QPX in the 13 °C treatment. Labyrinthulomycete phylogenetic composition of shell swab samples was similar between the two treatments, while in seawater, more oblongichytrids were found in the 20 °C treatment.

## 4. Discussion

*M. quahogii* can be found both within its host and in the surrounding habitat, even when there is not a QPX disease outbreak. The occurrence of QPX disease appears to depend on host–pathogen–environment interactions; how *M. quahogii* moves into and out of its hard clam host and the stability of the interaction between host and pathogen are not well understood. Based on available data, we hypothesized that *M. quahogii* release from naturally infected hard clams is common, regardless of temperature, and that *M. quahogii* is a long-term resident of the hard clam microbiota, characteristic of commensalism with the hard clam, and performed a microcosm experiment to test these hypotheses. The experiment used filtered, UV-sterilized seawater so that the only *M. quahogii*—and other labyrinthulomycetes—detected must have been introduced with the hard clams.

This is the first study to examine *M. quahogii* in hard clam pallial fluid of cultured hard clams from a QPX-enzootic site (Barnstable, MA, USA). Although the hard clams used in this experiment were not heavily infected with QPX disease (low tissue QPX WP representing light-intensity infections), there was a significant reduction in QPX load in tissue at 20 °C (Figure 1; Supplementary Table S2), which may represent clam healing due to impact of temperature on hard clam immunity and QPX disease development [25,26,28]. Our data confirm the release of *M. quahogii* from infected hard clams. Contrary to our hypothesis, *M. quahogii* was only detected in seawater when naturally infected live hard clams were subjected to the low-temperature treatment at 13 °C (Figure 3 and Figure 4; Supplementary Table S4). Similarly, *M. quahogii* was never detected in shell swab samples at 20 °C but was detected at 13 °C (Figure 4). The detection of *M. quahogii* in shell swabs is consistent with observations from the field, where *M. quahogii* has been detected from marine invertebrate shell biofilms, including the hard clam [23]. The confirmation of release of *M. quahogii* from hard clams supports that *M. quahogii* detected in the environment by past studies may represent *M. quahogii* released from infected hard clams in locations containing hard clam populations; however, it remains unknown if the release of *M. quahogii* from hard clams comes from *M. quahogii* residing in tissues, on the shell, and/or discharge from pallial fluid, particularly given the high prevalence and concentration of *M. quahogii* in pallial fluid (Figure 1 and Figure 2; Supplementary Tables S2–S4). Whether detection of *M. quahogii* outside hard clams at 13 °C indicates that release only occurs at 13 °C or that *M. quahogii* cannot maintain itself outside hard clams at 20 °C requires further study.

*M. quahogii* was found in 82% of hard clam pallial fluid samples after 9 weeks in a laboratory microcosm using sterile seawater (Figure 1 and Figure 4), suggesting that *M. quahogii* is a more permanent resident of the hard clam pallial fluid microbiota, representing a stable, long-term, likely commensal relationship. Moreover, this study supports a unique or host-specific relationship between *M. quahogii* and the hard clam, as no other thraustochytrids were identified as part of the hard clam microbiota (Figure 5). Of all the labyrinthulomycete groups, thraustochytrids tend to form host- or substrate-specific associations with marine animals, which are thought to be mutualistic or commensal in nature [10,11,12]. Although no other thraustochytrids were identified in this study as part of the hard clam microbiota, it is possible that other thraustochytrids may also be components of the hard clam microbiota. For example, a thraustochytrid, dubbed “C9G,” was isolated from the gills of a Canadian hard clam infected with QPX disease and was shown to be phylogenetically related to *M. quahogii* [35]. Interestingly, C9G lacked the characteristic mucus production (virulence factor) that makes *M. quahogii* unique amongst the thraustochytrids and provides protection from hard clam hemocytes [2].

*M. quahogii* was always present in the hard clam host—in either tissue or pallial fluid—with the exception of one hard clam sample (Tank #10, 13 °C), in which *M. quahogii* was not detected in either tissue or pallial fluid, but was detected in seawater (Figure 4 and Supplementary Table S4). The “high-low” relationship found between *M. quahogii* in hard clam mantle tissue and pallial fluid at the individual clam level in this microcosm experiment using MA cultured hard clams (Figure 2) was also observed in wild hard clams of New York [16]. While we cannot rule out that hard clam QPX tissue infections are the source of *M. quahogii* detected in hard clam pallial fluid or mesocosm seawater in this experiment, the field survey of wild hard clams of New York revealed that *M. quahogii* was present and abundant in pallial fluid from hard clams that were not infected with QPX disease (negative by qPCR diagnostic assay) [16]. These observations suggest that QPX-infected hard clam tissue is not the source of *M. quahogii* detected in hard clam pallial fluid. Although speculative, it is also possible that multiple strains of *M. quahogii* exist within a given environment, which may have preferential microhabitats within the environment or even the hard clam host (e.g., tissue vs. pallial fluid). In hard clam pallial fluid, *M. quahogii* may colonize mucosal secretions covering the soft body or use pseudofeces as a substrate for growth, as pseudofeces are formed and transit throughout the pallial cavity. In fact, past studies have shown that pseudofeces harbor *M. quahogii* [20,27]. However, this study shows that the presence of *M. quahogii* in the pallial cavity is not dependent on pseudofeces (Table S3), further underlining a possible colonization of the surface of clam soft tissues (mantle, gills, etc.).

Phylogenetic analyses of labyrinthulomycete PCR products from hard clam pallial fluid suggest that *M. quahogii* was the dominant labyrinthulomycete at 20 °C (100% of amplifiable samples), while at 13 °C, aplanochytrids were more dominant (64%). In addition to aplanochytrids in the 13 °C treatment, hard clam pallial fluid also harbored an oblongichytrid and an unidentifiable (but likely aplanochytrid or oblongichytrid based on the phylogenetic analysis; not shown) labyrinthulomycete. In seawater and shell swab samples, aplanochytrids were dominant, followed by oblongichytrids, which were particularly abundant in seawater of the 20 °C treatment (Figure 5). Additionally, *M. quahogii* was found in one seawater sample at the 13ºC with approximately equal contribution to the mixed labyrinthulomycete PCR product. This was from Tank #6, in which *M. quahogii* was consistently detected in seawater beginning at 2 weeks (Figure 3). The hard clam housed in this tank had the third highest concentration of *M. quahogii* in tissue and was BLD in pallial fluid, supporting the theory that an immune response by the hard clam (likely via mucosal immune effectors; discussed in [16]) can purge *M. quahogii* from the pallial cavity once *M. quahogii* breaches host barriers and invades the tissue [16]. The LABY-A/LABY-Y primer pair used in this analysis is specific for labyrinthulomycetes but is biased against many taxa and is notably less effective at detecting *M. quahogii* than the QPX-specific qPCR and nqPCR assays (Figure 4 and Figure 5).

Since the experimental MA hard clams were “purged” and housed in initially sterile seawater, the only source of labyrinthulomycetes detected later would be from the hard clam microbiota, and different labyrinthulomycetes (as well as other microbes) introduced with the hard clams may respond differently to the microcosm growth conditions. These could represent common associates of the hard clam or transient inhabitants of the microbiota inside the hard clam or living on the hard clam as biofilms. In the field study of wild hard clams from New York, *M. quahogii* was the dominant labyrinthulomycete in hard clam pallial fluid samples [16]; however, in this microcosm experiment using MA hard clams, there was a difference in labyrinthulomycete composition of pallial fluid between the two temperature treatments (Figure 5). The high prevalence of aplanochytrids in hard clam pallial fluid at 13 °C may represent enrichment of aplanochytrids by the mesocosm habitat, in combination with reduced pumping rates due to low-temperature stress on the hard clam [25]. From the perspective of *M. quahogii*, the lower temperature treatment may limit *M. quahogii’s* mucus production and ability to stay within the pallial fluid of the clam; this scenario seems the most probable and is further discussed below. The differences detected in *M. quahogii* prevalence and labyrinthulomycete assemblages between the two temperatures could also reflect a mechanism by the hard clam, labyrinthulomycete, or an interaction between the two. For example, *M. quahogii* could have been released from hard clams in both temperature treatments but only survived well enough to be detected in seawater at 13 °C because of a predator or some other limitation at 20 °C (i.e., environmental conditions determined *M. quahogii* detection limits). Alternatively, *M. quahogii* may have only been released at 13 °C because the clam produces inhibitory compounds at 20 °C that it does not at 13 °C, representing a clam immune response or regulation of “foreign invaders”.

The observation that *M. quahogii* is commonly found in hard clam pallial fluid and in what appears to be a stable, long-term relationship implies that *M. quahogii* has a mechanism to maintain itself in the pallial fluid, which warrants further investigation. Most labyrinthulomycetes possess ectoplasmic nets (EN), which serve to anchor vegetative (non-motile) cells to substrates for osmoheterotrophic nutrition [10]; however, it is unclear whether or not *M. quahogii* produces EN [8]. Instead, *M. quahogii* uniquely produces copious amounts of gel-like mucus in culture, hence its name [8], which may serve to “attach” or “stick” vegetative cells to hard clam pallial organs, reducing the potential for expulsion by hard clam pumping. In culture, the production of mucus by *M. quahogii* increases with increasing temperature up to 24 °C [36], which may account for the observed differences in the microcosm experiment between the 20 °C (100% QPX-positive pallial fluid samples) and 13 °C treatment (64% QPX-positive pallial fluid samples) (Figure 1). This could also explain the lack of *M. quahogii* identified in seawater and shell swab samples in the 20 °C treatment (Figure 3 and Figure 4). In addition to a mechanism to maintain itself in pallial fluid, *M. quahogii* must also have evolved to circumvent defense factors of pallial fluid, which have been implicated to be the first line of defense against pathogens and foreign invaders in bivalves [37,38,39]. In oysters, past studies have confirmed the presence of humoral defense factors in pallial fluid [37] and pallial mucus has been shown to regulate pathogenesis of the oyster parasite, *Perkinsus marinus* [38,40], suggesting pallial fluid and its associated mucus play an important role in bivalve defense. Yet, the role of pallial fluid and its potential defense-related properties have not been investigated in the hard clam in relation to *M. quahogii* or QPX disease. We can also speculate that the release of *M. quahogii* may be dependent on the hard clam host. Since QPX disease development is thought to occur under low temperature [25,26], the release of *M. quahogii* at low temperature may occur when infections are active, as opposed to the high temperature, which promotes clam healing; as QPX infection in tissue becomes more severe, the hard clam may enhance the production of mucosal immune effectors and effectively purge *M. quahogii* from the pallial cavity. Another possible explanation for the reduction of *M. quahogii* in hard clam pallial fluid and increased detection in seawater at 13 °C is changes to hard clam mucosal properties, such as reduced viscosity, which could result in enhanced flushing of pallial fluid mucus and its microbial inhabitants, like *M. quahogii*. Our understanding of the potential release of *M. quahogii* from pallial fluid is further hampered by the lack of knowledge on bivalve pallial fluid, such as the turnover time, microbial composition, and antimicrobial properties, including the factors that influence these aspects.

While the transmission mechanism of QPX disease remains unknown, QPX disease has successfully been transmitted to naïve hard clams by cohabitation with naturally infected hard clams [21]. It is also unknown whether the *M. quahogii* released from hard clams in this study are infectious. Additionally, factors affecting the relationship between *M. quahogii* detected in hard clam tissue and *M. quahogii* detected in pallial fluid remain unknown. Although pathogen reservoirs are usually considered to be secondary hosts or substrates in the environment, this study suggests that pallial fluid represents a stable *M. quahogii* “reservoir” within its primary host. In this light, *M. quahogii* could exist and concentrate in pallial fluid, perhaps as a commensal using pseudofeces and other organic matter for osmoheterotrophic nutrition, until it can opportunistically penetrate, breach host barriers, and infect the clam tissue resulting in QPX disease, as a function of host immune status or changes in host–microbe–environment interactions. Given the close and stable relationship between *M. quahogii* and the hard clam, excluding *M. quahogii* from hard clam cultivation is probably not possible; therefore, disease management strategies have to focus on improving clam stocks via selective breeding as well as mitigating the underlying causes of QPX disease (e.g., factors that result in immunocompromised hard clams) and better consideration of environmental conditions that favor resistance.

## 5. Conclusions

*M. quahogii* was commonly found in hard clam pallial fluid, even after 9 weeks in the lab, suggesting pallial fluid is a stable environmental reservoir of *M. quahogii* within its primary host and that *M. quahogii* is not a transient component of the hard clam microbiota. Besides *M. quahogii*, aplanochytrids and oblongichytrids were identified, likely representing common components of the hard clam microbiota. The release of *M. quahogii* from naturally infected live hard clams was confirmed, although released *M. quahogii* was only detected in seawater and shell biofilms at 13 °C and never detected at 20 °C, illustrating the interactions between host, pathogen, and the environment. It remains unknown if released *M. quahogii* from hard clam tissue or pallial fluid into the environment represents “infectious” cells. Overall, results support a host-specific relationship between *M. quahogii* and the hard clam, with *M. quahogii* as a commensal or avirulent member of the hard clam microbiota until perturbations in host immunity or the host–microbe–environment relationship result in virulence and pathogenesis, supporting its classification as a commensal, opportunistic pathogen.

## Data Availability

Data are contained within the article and supplementary materials.

## Supplementary Materials

The following supporting information can be downloaded at: https://www.mdpi.com/article/10.3390/microorganisms12020241/s1, Table S1: Intensity scale used to determine *M. quahogii* (=QPX) weighted prevalence in hard clam mantle tissue and pallial fluid based on the QPX qPCR assay. Scales differ due to different detection limit of the two assays; Table S2: Descriptive statistics of *M. quahogii* (=QPX) prevalence, weighted prevalence (WP), and concentration (copies/mg or ml) for hard clam mantle tissue and pallial fluid samples, assayed by qPCR; Table S3: Descriptive statistics of *M. quahogii* (=QPX) prevalence, weighted prevalence (WP), and concentration (copies/mL) for hard clam pallial fluid (PF) samples with or without pseudofeces, assayed by qPCR; Table S4: *M. quahogii* (=QPX) concentration (copies/mg or ml) by treatment for hard clam mantle tissue, pallial fluid, microcosm seawater (9 week sample only) and hard clam shell biofilms (swabs) determined by qPCR or nqPCR assays; File S1: QPX_Microcosm_sequences_alignment.fas; File S2: QPX_Microcosm_sequences_alignment_key.xlsx.
