# Supplementary material for: A Microcosm Experiment Reveals the Temperature-Sensitive Release of Mucochytrium quahogii (=QPX) from Hard Clams and Pallial Fluid as a Stable QPX Reservoir
Source: Microorganisms. 2024 Jan 24;12(2):241. doi: 10.3390/microorganisms12020241 (PMC10892119; doi:10.3390/microorganisms12020241)
Supplement: Supplementary file 1 [file microorganisms-12-00241-s001.zip › microorganisms-2775856-supplementary tables.docx]

**Supplemental Tables**

**Table S1:** Intensity scale used to determine *M. quahogii* (=QPX) weighted prevalence in hard clam mantle tissue and pallial fluid based on the QPX qPCR assay. Scales differ due to different detection limit of the two assays.

| **Intensity** | **Tissue**  **QPX copies/mg** | **Pallial Fluid**  **QPX copies/ml** |
| --- | --- | --- |
| 0 = None | 0 = negative | 0 = negative |
| 1 = Rare | < 75 = BLD | < 500 = BLD |
| 2 = Light | 440 | 880 |
| 3 = Mild | 2,200 | 2,200 |
| 4 = Moderate | 11,000 | 11,000 |
| 5 = Heavy | 55,000 | 55,000 |
| 6 = Severe | > 55,000 | > 55,000 |

BLD = below limit of detection

**Table S2:** Descriptive statistics of *M. quahogii* (=QPX) prevalence, weighted prevalence (WP), and concentration (copies/mg or ml) for hard clam mantle tissue and pallial fluid samples, assayed by qPCR.

| **Sample Type/Statistic** | | **Pre-Exp.** | **Post 13°C** | **Post 20°C** | **Post ALL** |
| --- | --- | --- | --- | --- | --- |
| **Tissue** | Prevalence (%) | 100 | 91 | 100 | 95.5 |
|  | WP | 1.88 | 1.55 | 1.82 | 1.69 |
|  | Mean ± SD | 198 ± 107 | 259 ± 104 | 105.5 ± 20 | 182.25 ± 62 |
|  | Minimum | 88.7 | 95.9 | 81.4 | 81.4 |
|  | Maximum | 464.9 | 389.3 | 134.2 | 389.3 |
| **Pallial** | Prevalence (%) | n/a | 64 | 100 | 82 |
|  | WP | n/a | 1.91 | 2.73 | 2.32 |
|  | Mean ± SD | n/a | 28,468 ± 57,551 | 2,398 ± 1,453 | 15,433 ± 29,502 |
|  | Minimum | n/a | 1,070 | 995 | 1,033 |
|  | Maximum | n/a | 131,377 | 5,436 | 68,406 |

SD = standard deviation; n/a = not applicable

**Table S3:** Descriptive statistics of *M. quahogii* (=QPX) prevalence, weighted prevalence (WP), and concentration (copies/ml) for hard clam pallial fluid (PF) samples with or without pseudofeces, assayed by qPCR.

| **Statistic** | **PF with Pseudofeces (n=5)** | **PF without Pseudofeces (n=17)** |
| --- | --- | --- |
| QPX Prevalence Total Positive | 100% | 76.46% |
| QPX Prevalence Positive | 80% | 58.82% |
| QPX Prevalence BLD | 20% | 17.64% |
| QPX Prevalence Negative | 0% | 23.54% |
| Weighted Prevalence (WP) | 2.8 | 2.2 |
| Mean ± Standard Deviation | 2,156 ± 1,070 | 15,316 ± 40,816 |
| Minimum Concentration | 995.36 | 268.32 |
| Maximum Concentration | 3,588.02 | 131,376.79 |

BLD = below limit of detection

**Table S4:** *M. quahogii* (=QPX) concentration (copies/mg or ml) by treatment for hard clam mantle tissue, pallial fluid, microcosm seawater (9 week sample only) and hard clam shell biofilms (swabs) determined by qPCR or nqPCR assays.

| **Treatment** | **Tank #** | **Tissue**  **copies/mg** | **Pallial Fluid**  **copies/ml** | **Seawater**  **copies/ml** | **Shell**  **copies/swab** |
| --- | --- | --- | --- | --- | --- |
| 13°C | 1 | 96 | 1070 | 0 | 0 |
|  | 2 | 277 | 5,438 | 3.1 | 0 |
|  | 3 | 313 | 2,025 | 0 | 0 |
|  | 4 | 389 | BLD | 0 | 0 |
|  | 5 | BLD | 2,428 | 0.9 | 1,504 |
|  | 6 | 301 | BLD | 97.6 | 3,818 |
|  | 7 | BLD | 0 | 0.7 | 0 |
|  | 8 | 297 | 0 | 0.5 | 0 |
|  | 9 | BLD | 131,377 | 1.3 | 1,546 |
|  | 10 | 0 | 0 | 0.3 | 0 |
|  | 11 | 138 | 0 | 0 | 346 |
| 20°C | 12 | BLD | 2,017 | 0 | 0 |
|  | 13 | 81 | 995 | 0 | 0 |
|  | 14 | BLD | 3,588 | 0 | 0 |
|  | 15 | 101 | 5,436 | 0 | 0 |
|  | 16 | 123 | 1,477 | 0 | 0 |
|  | 17 | 134 | BLD | 0 | 0 |
|  | 18 | 86 | 1,907 | 0 | 0 |
|  | 19 | 126 | 2,333 | 0 | 0 |
|  | 20 | 118 | BLD | 0 | 0 |
|  | 21 | 99 | 1,430 | 0 | 0 |
|  | 22 | 81 | 268 | 0 | 0 |

BLD = below the limit of detection (positive, but non-quantifiable)

**Supplementary Files**

**Supplementary File S1:** QPX_Microcosm_sequences_alignment.fas

**Supplementary File S2:** QPX_Microcosm_sequences_alignment_key.xlsx

Supplementary File S1 is the nucleotide sequence alignment of LABY-A/LABY-Y PCR products with labyrinthulomycete reference sequences curated from NCBI GenBank. There is a total of 134 sequences, comprised of 65 labyrinthulomycete reference sequences and 69 microcosm sequences. Of the microcosm sequences, 30 were original sequences that had clean chromatograms; the remaining 28 original sequences were deconvoluted with BCV into 39 ‘clusters.’ Microcosm sequences are named “SGY###” and can be identified using the key in Supplementary File S2.
